# The Effects of Propofol and Thiopental on Nitric Oxide Production and Release in Erythrocytes

**DOI:** 10.3390/medicina61050841

**Published:** 2025-05-02

**Authors:** Ulku Arslan, Pinar Ulker, Ahmet Yildirim, Melike Cengiz, Murat Yilmaz, Ayse Gulbin Arici, Emel Gunduz, Ali Sait Kavakli, Arzu Hizay, Oguzhan Arslan, Zeynep Yasemin Tavsanoglu, Nihal Ozturk

**Affiliations:** 1 Department of Anesthesiology and Reanimation, Akdeniz University Faculty of Medicine, 07070 Antalya, Turkey; drulkuarslan@gmail.com (U.A.); melikecengiz@yahoo.com (M.C.); muryigit@yahoo.com (M.Y.); gulbinarici@akdeniz.edu.tr (A.G.A.); dregunduz@hormail.com (E.G.); 2 Department of Physiology, Akdeniz University Faculty of Medicine, 07070 Antalya, Turkey; ahmetyildirim398@gmail.com; 3 Department of Anesthesiology and Reanimation, Istinye University Faculty of Medicine, 34396 Istanbul, Turkey; alisaitkavakli@hotmail.com; 4 Department of Anatomy, Akdeniz University Faculty of Medicine, 07070 Antalya, Turkey; hizayarzu@gmail.com; 5 Department of Pharmacology, Akdeniz University Faculty of Medicine, 07070 Antalya, Turkey; droguzhanarslan@gmail.com; 6 Department of Anesthesiology and Reanimation, Kocaeli City Hospital, 41000 Kocaeli, Turkey; zeynepyaseminyilmaz@gmail.com; 7 Department of Biophysics, Akdeniz University Faculty of Medicine, 07070 Antalya, Turkey; nozturk@akdeniz.edu.tr

**Keywords:** anaesthetic drug, erythrocyte, nitric oxide, propofol, thiopental

## Abstract

*Background*: Hypotension is a common adverse effect associated with the use of propofol and sodium thiopental. The objective of this study was to examine the impact of thiopental and propofol on erythrocyte (RBC) nitric oxide (NO) synthase activity and RBC-mediated NO release. *Methods*: A prospective, interventional in vitro trial. Male patients aged between 18 and 45 years with a classification of American Society of Anesthesiologists (ASA) class I, defined as healthy individuals, were included in this study. Venous blood samples (20 mL) were obtained from patients who met the inclusion criteria. Measurements were performed using the specific fluorescent probes for NO and calcium (Ca^2+^). Propofol and sodium thiopental were added to the suspensions at doses of 100, 250, 500, and 1000 μM and incubated for 30 min. All suspensions were proceeded to flow cytometric analysis. Nitrite/nitrate concentration was measured in the supernatant of RBC suspensions after centrifugation. RBC deformability and aggregation were measured by laser diffraction analysis using an ektacytometer. The primary outcome was to evaluate the effects of sodium thiopental and propofol on RBC-NOS activity. *Results*: Sodium thiopental caused significant increase in intracellular NO concentrations at all doses studied (*p* < 0.001). Importantly, the intracellular NO concentration increment was positively correlated with sodium thiopental concentration in the suspensions. The presence of L-N-acetylmethyl-arginine in the experimental medium abolished NO production in RBCs in response to sodium thiopental. Sodium thiopental caused increased nitrite and nitrate levels in the suspension medium in a dose-dependent manner. Incubation with thiopental caused an increase in intracellular free Ca^+2^ levels while propofol induced no change. Sodium thiopental and propofol caused significant decrement in RBC aggregation. *Conclusions*: This study presents the initial evidence of augmented RBC-mediated NO production and release in response to sodium thiopental administration. In contrast to the effects observed with sodium thiopental, our results demonstrated that propofol had no impact on RBC-mediated NO production.

## 1. Background

Intravenous anaesthetic agents are widely used for the rapid induction and maintenance of general anaesthesia in many perioperative situations where sedation is required, as well as in intensive care units (ICUs) to improve patient comfort, facilitate care, and tolerate mechanical ventilation. Propofol and sodium thiopental (ST) are two commonly used intravenous anaesthetic agents. Before the introduction of propofol, ST was the most commonly used intravenous anaesthetic agent for induction of anaesthesia [1]. In recent years, propofol has become the most preferred intravenous anaesthetic agent for induction and maintenance of anaesthesia and is widely used for short-term sedation in intensive care units and interventional procedures outside the operating room [2]. However, ST is still widely used for rescue treatment of refractory status epilepticus and intracranial hypertension in ICUs in many countries outside the United States [3,4].

Hypotension is frequently encountered during the use of these commonly used intravenous anaesthetic agents (ST and propofol). This is thought to be a consequence of their effects on the sympathetic nervous system, myocardial contractility, or vascular tone. The reduction in systemic arterial blood pressure following ST and propofol induction is primarily attributable to peripheral vasodilation [5,6,7]. However, the precise mechanisms behind the peripheral vasodilator effects of both drugs, as well as the specific mediators involved, remain unclear. Prior research has emphasised the potential connection between systemic vasodilation and the elevated production of nitric oxide (NO), a tiny gaseous and lipophilic molecule that plays a crucial role in the regulation of vascular homeostasis and haemoregulation [8,9,10,11]. NO is synthesised from L-arginine in a reaction catalysed by nitric oxide synthase (NOS). Three types of NOS isoforms have been defined as neuronal NOS, inducible NOS, and endothelial NOS (eNOS or NOS3) [12]. It is known that NO produced by eNOS or NOS3 in endothelial cells has an important role in the regulation of vascular tone and haemodynamics [13]. Previous studies have reported that NO is responsible for both the vasodilator effects associated with ST and propofol [14,15]. Similar to NO production in endothelial cells, it is currently known that erythrocytes (RBCs) have a nitric oxide synthase (RBC-NOS), which is an enzyme known to enhance the production of NO. Furthermore, prior research has demonstrated the crucial roles of RBC-NOS enzyme activity in regulating physiological processes such as circulating blood flow, blood pressure, and myocardial function. However, it is not known whether ST and propofol, which have been shown to produce NO-mediated vasodilation in previous scientific studies, affect RBC-NOS-mediated NO release. It is of significant importance to conduct studies that facilitate the development of new methods and strategies to prevent the hypotensive effects of these drugs. Therefore, the aim of this in vitro study was to investigate the effects of ST and propofol on RBC-NOS activity and RBC-mediated NO release.

## 2. Methods

### 2.1. Study Design and Ethical Approval

This was a prospective, interventional in vitro study conducted at the Departments of Physiology and Anaesthesiology and Reanimation, Faculty of Medicine, Akdeniz University. The sample was collected from 1 March 2024 to 1 July 2024. The current study was carried out in accordance with the Declaration of Helsinki and approved by the Ethics Committee of Akdeniz University Faculty of Medicine, Antalya, Turkey (chairperson: Professor Doctor Mehmet Rifki Aktekin; approval no.: TBAEK-115; dated: 29 February 2024). In addition, this trial was registered prior to patient enrolment at clinicaltrials.gov (no.: NCT06485388; principal investigator: Ulku Arslan; date of registration: 17 March 2024). Written informed consent was obtained from all subjects participating in the trial.

### 2.2. Participants

This study included male patients aged 18–45 years with an American Society of Anesthesiologists (ASA) class I classification, defined as a normal, healthy individual without any disease or systemic problem other than surgical pathology that does not cause a systemic disorder. The patients were recruited from the Department of Anaesthesiology and Reanimation outpatient clinic and were patients admitted for preoperative preparation. It is established that oestrogens enhance the production of NO in RBCs by stimulating the phosphorylation of the RBC-NOS enzyme [16]. Consequently, female patients, patients with an ASA class > 1, patients with a known ST/propofol allergy, and patients outside the age range of 18–45 years were excluded from this study. This study was carried out by performing in vitro experiments on venous blood samples from patients who had not taken any medication for 15 days prior to blood sampling.

### 2.3. Outcome

The primary outcome was to evaluate the effects of ST and propofol on RBC-NOS activity. The secondary outcome was to examine the effects of ST and propofol on RBC-mediated NO release and erythrocyte haemorheology.

### 2.4. Blood Samples and Preparation of RBC Suspensions

Venous blood samples (20 mL) obtained from patients who met the inclusion criteria were anticoagulated with sodium heparin (15 IU/mL). Leukocytes were removed by centrifuging 20 mL of blood through 20 mL of a polysucrose (60 gr/L) and sodium diatrizoate (167 gr/L) solution (Histopaque1119; Sigma Chemical Co., St. Louis, MO, USA) in a 50 mL polypropylene tube at 700× *g* for 30 min. RBC pellet was washed three times with calcium-and magnesium-free phosphate buffered saline (PBS, Sigma Chemical Co., St. Louis, MO, USA. 290 mOsm/kg, pH = 7.4) and resuspended in HEPES buffer (125 mmol/L NaCl, 3 mmol/L KCl, 1 mmol/L MgCl_2_, 2 mmol/L CaCl_2_, 16 mmol/L HEPES, 1.2 mmol/L sodium phosphate, and 10 mmol/L glucose, pH 7.4) at a haematocrit of 0.01 l/L. All procedures were conducted at room temperature (RT; 20 ± 2 °C, 68 ± 4 °F), unless otherwise indicated.

### 2.5. Demonstration of NO Generation

Measurements were performed using a 4-amino-5-methylamino-20,70-difluorofluorescein diacetate (DAF-FM DA) fluorescent NO probe. Firstly, a stock solution was prepared by dissolving the DAF-FM DA in dimethylsulfoxide. Then, aliquots were stored at −20 °C until the day of experiment. RBC suspensions used for monitoring changes in intracellular NO concentration were incubated at room temperature with 5 µM DAF-FM DA for 30 min, then washed three times with HEPES, resuspended with the same buffer, and incubated for 15 min to allow de-esterification of DAF-FM. The optimal plasma concentration for the use of ST and propofol in clinical practice remains unclear. A fundamental principle in pharmacology asserts that the magnitude of the drug effect is directly proportional to the dosage administered. This principle constitutes the basis of anaesthetic titration, whereby the dosage is increased when an escalating drug effect is desired and decreased when a diminishing drug effect is sought. [17]. However, in recent years, the paradox of drug titration has come to the fore [17,18,19,20]. At the population level, there is a consensus that the observed correlation between drug concentration and outcome may not accurately represent the true relationship between drug concentration and its causal effect on outcome [18]. This emphasises the necessity for pharmacodynamic and pharmacokinetic studies to determine the optimal plasma concentration for pharmacological effects, taking into account the drug titration paradox. Moreover, it is broadly acknowledged that the relationship between ST concentration and pharmacological responses, encompassing neurological response, intracranial pressure, electroencephalography, and drug toxicity, is not robust [21,22]. Stoover et al. reported that the serum level of ST does not correlate well with dose and is not useful in predicting physiological symptoms such as depth of anaesthesia [23]. In conclusion, a wide range of propofol and ST concentrations were studied, recognising that the optimum plasma concentrations for propofol and ST remain uncertain and taking into account the doses used in previous documented studies [10,24,25,26]. Propofol and ST were added to the suspensions at doses of 100, 250, 500, and 1000 µM and incubated for 30 min at room temperature (Table 1). Based on the aim of the experiment, L-N-acetylmethyl-arginine (L-NAME, 1 mM) was added to the suspensions and incubated for 30 min prior to adding propofol and ST. After incubation procedures, all resuspensions were proceeded to flow cytometric analysis. Additionally, samples comprising solely erythrocyte suspension devoid of DAF-FM DA (negative samples) underwent flow cytometric analysis to substantiate the loading of DAF-FM DA. A total of 10,000 cells were analysed by flow cytometry for each sample, using a suspension of RBCs at a concentration of 1% (250 μL). Considering that the DAF-FM fluorescence signal may be dependent on the incubation time, the timing of flow cytometry analysis after treatment was the same for all samples.

### 2.6. Demonstration of Intracellular Calcium (Ca^+2^) Levels

Fluo-4/AM (F1241, Invitrogen, Carlsbad, CA, USA) was added to RBC suspensions at a dose of 1 µM and incubated with mild shaking for 30 min at 37 °C. RBCs were washed three times with PBS following the incubation period and then resuspended in HEPES buffer which was used to achieve 0.01 l/L haematocrit value. Propofol and ST were added to the suspensions at doses of 100, 250, 500, and 1000 µM and incubated for 30 min at room temperature. Then, all resuspensions were subjected to flow cytometric analysis. In the context of flow cytometric analysis, a negative reading was assigned a value of ‘1’, and the intracellular Ca^2+^ FL ratio was calculated by proportioning the irradiated values to their negative sample value.

### 2.7. Demonstration of Nitrite/Nitrate Levels

Nitrite/nitrate concentration was measured in the supernatant of RBC suspensions after centrifugation at 2700 RPM for 6 min. The supernatant was passed through a filter with a 10 kDa cut-off by centrifugation at 14,000 RPM for 120 min. The filtrate was incubated with nitrate reductase and cofactor for 3 h at room temperature to convert nitrate in the sample to nitrite. Total nitrite concentration, reflecting nitrate/nitrate concentration in the sample, was then calculated using absorbance measured at 540 nm after the reaction with Griess reagent. A commercial kit was used for nitrite/nitrate determination (780001, Cayman Chemical Co., Ann Arbor, MI, USA).

### 2.8. RBC Deformability Measurements

RBC deformability was measured as an elongation index (EI) at 37 °C for various fluid shear stresses by laser diffraction analysis using an ektacytometer (LORRCA, RR Mechatronics, Hoorn, The Netherlands). The fundamental principles of the system have been elucidated in detail in a previous publication [27]. Briefly, a low haematocrit suspension of RBCs in an isotonic viscous medium (4% polyvinylpyrrolidone 360 solution; MW 360 kDa) is sheared in a Couette system composed of a glass cup and a precisely fitting bob, with a gap of 0.3 mm between the cylinders. A laser beam is directed through the sample and the diffraction pattern produced by the deformed cells is analysed with a microcomputer. Based upon the geometry of the elliptical diffraction pattern, an EI is calculated as: EIs(L−W)/(LqW), where L and W are the length and width of the diffraction pattern. At a constant shear stress, EI increases with RBC deformability. EI values determined at the nine shear stress between 0.3–30 pascal (Pa) were used to calculate the shear stress required for half-maximal RBC deformation (SS1/2) using Lineweaver–Burk analysis as described elsewhere [28]. Briefly, shear stress–EI curves were linearized by plotting the reciprocal of EI as the function of the reciprocal of shear stress. The x-intercept of the line obtained by simple linear regression corresponds to the negative reciprocal value of shear stress causing SS1/2. Thus, increased SS1/2 values indicate impairment of RBC deformability.

### 2.9. RBC Aggregation Measurements

RBC aggregation was evaluated using the same ektacytometer (LORCA, RR Mechatronics, Hoorn, The Netherlands). Aggregation was determined by placing 1 mL of blood into the gap between the cup and bob. The sample was then sheared at 800 1/s for 10 s to disperse pre-existing aggregates. Following the 10 s period, shear was abruptly decreased to zero and the level of laser light reflected from the sample was digitally recorded for 120 s. A dimensionless aggregation index (AI) was derived from the shape of the light reflection–time curve; AI increases with enhanced RBC aggregation. All measurements were conducted at 37 °C.

### 2.10. Statistical Analysis

#### Sample Size

To determine the minimum sample size, a power analysis was performed using the G*Power software (version 3.1.9.7) for a repeated measure ANOVA test. The measurement level was assumed to be four, and the analysis was conducted with a two-sided alpha level (type I error) of 0.05, a power (1 − β) of 0.80, and a large effect size (f = 0.4). Based on the results, we determined the minimum sample size to be 10 patients. Leaving a 20% margin in order to eliminate possible losses during the course of study, we enrolled this study with 12 patients. We completed this study with 12 patients and carried out a post hoc power analysis, indicating an achieved power level of 88.7%.

### 2.11. Data Analysis

All the graphs were made using the Graph Pad Prism (version 9.5.1) software. The remaining analyses were calculated using SPSS version 18 statistical software (SPSS Inc, Chicago, IL, USA). Continuous variables were expressed as interquartile range (IQR) (median, minimum and maximum) and categorical variables were expressed as frequency (N) and percentages (%). Considering the sample size in our study as well as the data distribution, non-parametric tests were used. The data from the multiple dose groups were analysed using the Friedman’s test to identify any significant changes. If such changes were observed, the Wilcoxon signed-rank test was employed to compare the results of two observations. The Bonferroni correction was applied to account for multiple comparisons. A two-tailed *p* value of less than 0.05 was considered to indicate statistical significance. The EC50 of propofol and ST in each group was determined by a modification of Dixon’s up-and-down method [29] and defined as the mean cross-over midpoint in each group.

## 3. Results

### 3.1. Patient Characteristics

**Figure 1:**
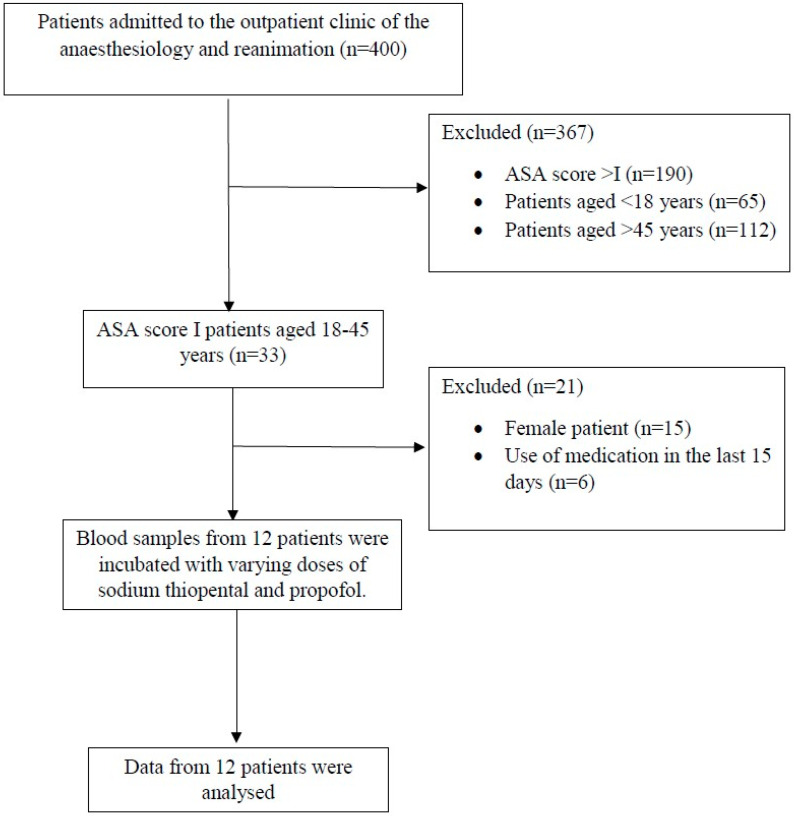
Flow diagram of this study.

A total of 400 patients admitted to the outpatient clinic of the Anaesthesiology and Reanimation Department were evaluated for this study. Data of 12 patients who met the inclusion criteria were analysed (Figure 1). Patient characteristics are presented in Table 2.

### 3.2. NO Generation from RBCs in Response to Thiopental and Propofol Incubation

**Figure 2:**
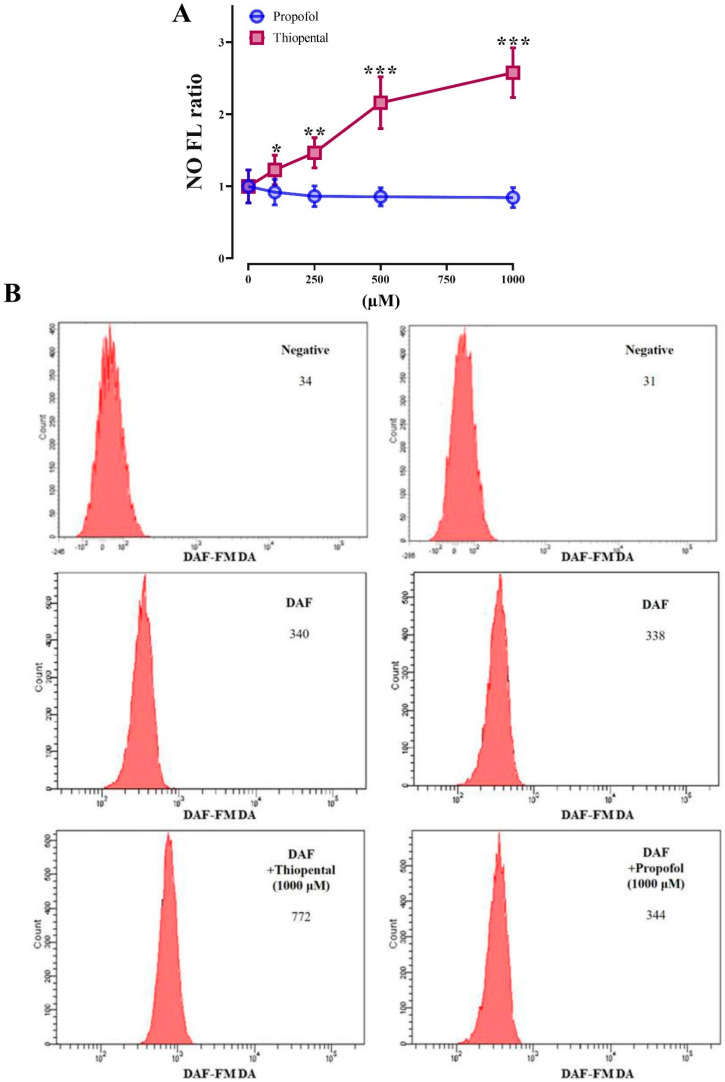
Percentage increase in NO concentration-related fluorescence of RBCs exposed to various levels of thiopental and propofol (**A**). An example of a flow cytometry result (**B**). Data presented are means ± SD. Error bars represent standard deviation. * *p* < 0.05, ** *p* < 0.01, *** *p* < 0.001, significantly different basal fluorescence levels; Friedman’s test followed by Bonferroni post hoc test. (*n* = 12).

**Figure 3:**
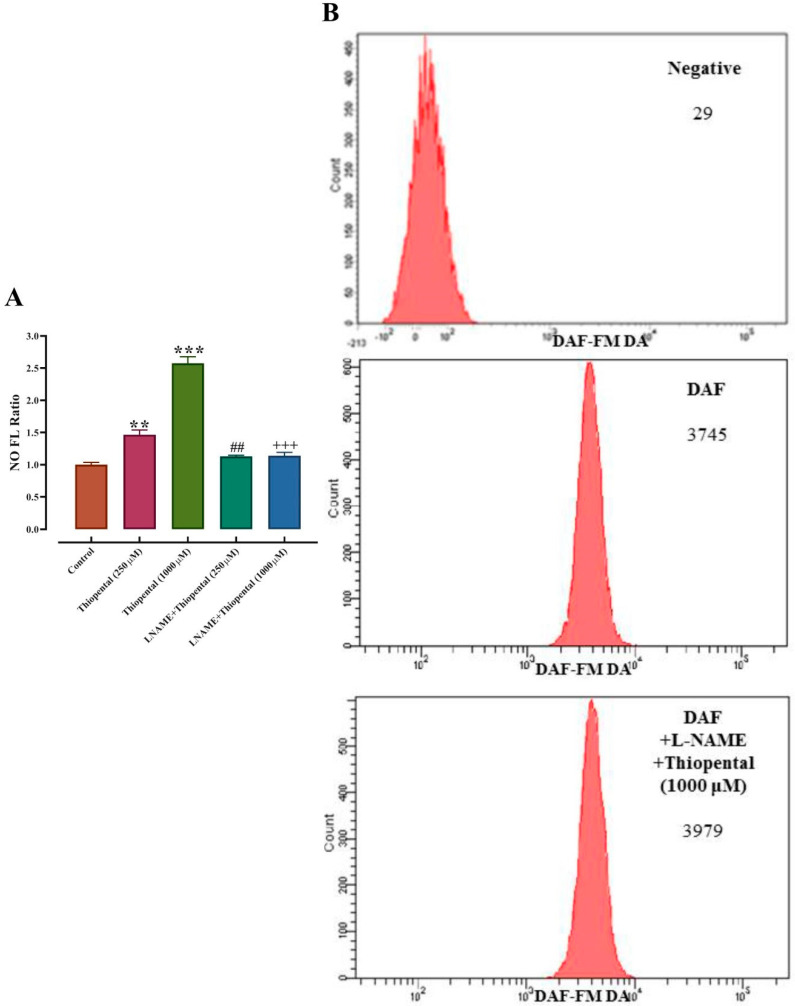
(**A**): Effects of L-NAME on thiopenthal-stimulated intracellular NO generation in RBCs. (**B**)**:** An example of a flow cytometry result. Data presented are means ± SEM. Error bars represent standard error of mean. ** *p* < 0.01, *** *p* < 0.001, difference from control; Friedman’s test followed by Bonferroni post hoc test. ## *p* < 0.01, difference from thiopental 250 µM, +++ *p* < 0.001, difference from thiopental 1000 µM; Wilcoxon signed-rank test. (*n* = 12).

Figure 2 represents percent increase in DAF-FMDA fluorescence intensity (indicative of NO production) in response to incubation of RBC suspensions with ST and propofol at doses of 100, 250, 500, and 1000 µM. ST caused statistically significant increases in intracellular NO concentrations at all administered doses. The increase in intracellular NO concentration was observed to be in proportion to the rise in ST concentration in the suspensions (Figure 2). However, in contrast to ST, propofol did not cause intracellular NO production in RBCs. The concentration of ST that increased NO production by 50% (EC50) was 415.8 ± 37.4 μM (109.9 ± 9.9 µg/mL). Our experiments were repeated in the presence of L-N-acetyl-methyl-arginine (L-NAME,1 mM), a non-specific NOS inhibitor, in order to prove that thiopental-mediated NO production in RBCs is related to RBC eNOS enzyme activation. As presented in Figure 3, the presence of L-NAME in the experimental medium abolished NO production in RBCs in response to ST treatment. Based on the data, these results indicate for the first time that ST stimulates NO generation in RBCs through NOS activation.

### 3.3. NO Output from Red Blood Cells in Response to Thiopental and Propofol Incubation

**Figure 4:**
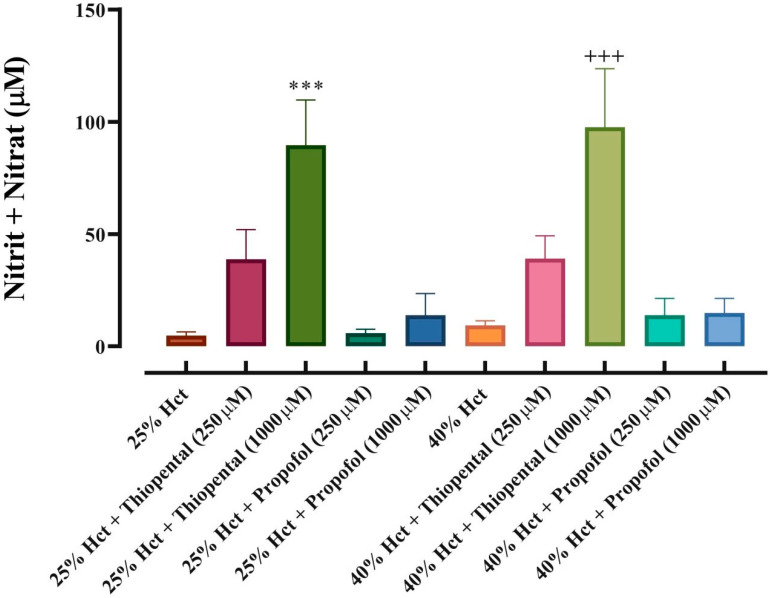
Nitrite and nitrate concentrations in supernatants of RBC suspensions. Data presented are means ± SEM. Error bars represent standard error of mean. *** *p* < 0.001, difference from 25% hct; Friedman’s test followed by Bonferroni post hoc test. +++ *p* < 0.001, difference from 40% hct; Friedman’s test followed by Bonferroni post hoc test. (*n* = 12).

In order to evaluate NO release in response to thiopental and propofol incubation from RBCs, nitrite and nitrate levels were measured in the supernatants of RBC suspensions (Figure 4). In these analyses, the dose-dependent effects of ST and propofol were evaluated separately in RBC suspensions prepared at 25% and 40% haematocrit, with the aim of determining whether there were any differences in the observed effects due to the variability in RBC concentration. Consistent with flow-cytometry measurements, ST caused increased nitrite and nitrate levels in the suspension medium in a dose-dependent manner. This increase was found to be statistically significant at a dose of 1000 µM. Although the increase was more pronounced in the 40% haematocrit group than in the 25% group, no statistical difference was observed between the two groups. In contrast, propofol did not cause an increase in nitrite/nitrate concentrations in RBC suspensions at any administered doses. Consistent with flow cytometric analysis, nitrite/nitrate measurements also demonstrated that, while ST caused NO generation and output from RBCs, propofol had no effect on RBCs regarding NO generation capacity.

### 3.4. Intracellular Ca^+2^ Concentrations

**Figure 5:**
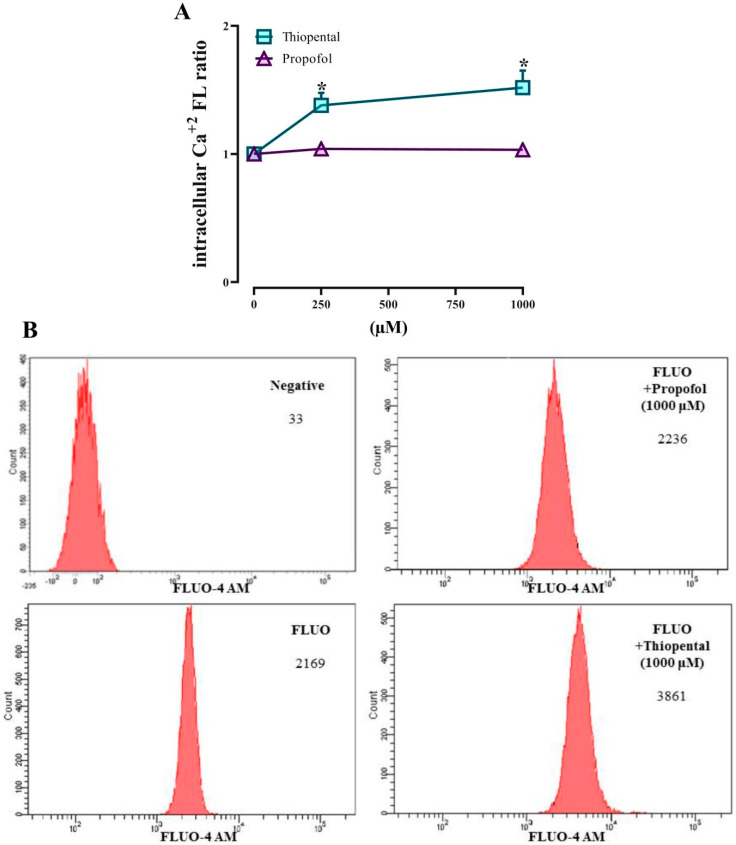
(**A**): Change in intracellular free-calcium-concentration-related fluorescence in Fluo-4 loaded RBCs. (**B**): An example of a flow cytometry result. Data presented are means ± SEM. Error bars represent standard error of mean. * *p* < 0.05, significantly different basal fluorescence levels; Friedman’s test followed by Bonferroni post hoc test. (*n* = 12).

Increment in intracellular Ca^+2^ levels causes calmodulin binding to NOS protein, which in turn leads to activation of NOS via detaching it from the membrane. In this regard, we investigated intracellular Ca^+2^ changes in response to administering ST and propofol to RBC suspensions. Figure 5 represents RBC intracellular calcium changes. ST incubation for 30 min caused increment in intracellular free Ca^+2^ levels at doses of 250 and 1000 µM. Incubation of RBCs with propofol did not cause any changes in intracellular free Ca^+2^ levels.

### 3.5. Haemorheological Analysis

**Figure 6:**
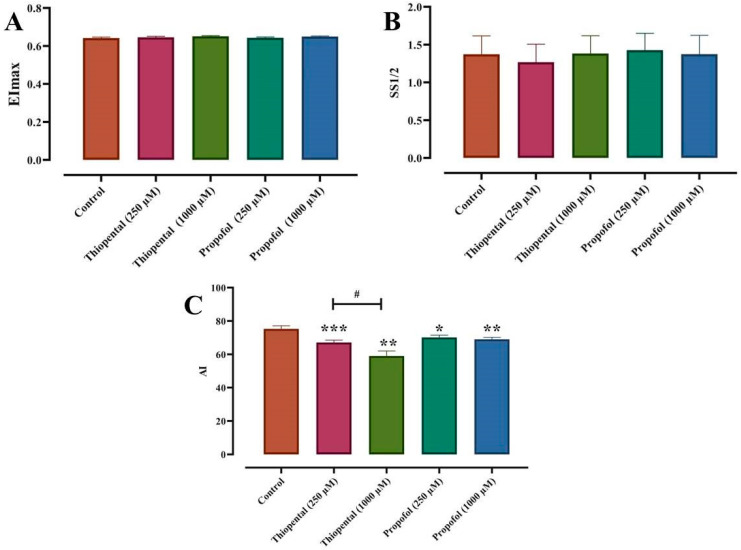
Propofol and thiopental decreased aggregation index (**C**) and caused no change in RBC deformability (**A**,**B**). * *p* < 0.05, ** *p* < 0.01, *** *p* < 0.001, difference from control; # *p* < 0.05: difference between thiopental doses of 250 and 1000 µM; Friedman’s test followed by Bonferroni post hoc test. Data presented are means ± SEM. Error bars represent standard error of mean. (*n* = 12).

ST and propofol, at doses of 250 and 1000 µM, caused significant decrement in RBC aggregation measured in autologous plasma. Although the effect of ST on RBC aggregation was dose-dependent, propofol exhibited a consistent effect across all administered doses. In addition, ST and propofol did not affect RBC deformability as demonstrated by the elongation index (EI) and the shear stress required for half-maximal RBC deformation (SS1/2) (Figure 6).

## 4. Discussion

This study provides the first evidence of increased RBC-mediated NO production and release in response to ST administration. This response was inhibited in the presence of 1 mM L-NAME, a non-specific competitive inhibitor of NOS, strongly suggesting that the response is related to the enzymatic generation of NO. It has also been shown that intracellular calcium elevation contributes to ST-mediated NOS activation in RBCs, suggesting a calcium dependence of NOS activation in RBCs. Nitrite/nitrate concentrations measured in the suspension medium also demonstrated that NO produced in RBCs was released in response to ST stimulation. In contrast to ST, our results revealed that propofol had no effect on RBC-mediated NO production. It was also demonstrated that both ST and propofol caused a decrease in the RBC aggregation index but had no effect on RBC deformability.

In clinical practice, hypotension is frequently encountered after administration of anaesthetic agents. Even a slight decrease in blood pressure in the intraoperative period is associated with serious complications such as acute kidney injury, major adverse cardiac events, and stroke [30,31]. It is crucial to stabilise hemodynamic parameters in order to prevent the occurrence of these serious complications. Although there is a plethora of suggestions in the literature regarding the administration techniques of these agents with the aim of optimising hemodynamics, there is yet no definitive solution available. Approximately 20–30% of patients still develop hypotension after induction of anaesthesia [32]. A definitive solution to preventing hypotension and related complications will only be possible once the mechanism is understood. ST and propofol are the most commonly used anaesthetic agents known to cause hypotension worldwide. Although hypotension is attributed to vasodilation and suppressive effects on myocardial contractility, sympathetic activity, baroreflex activity, and central nervous system activity, the relative potential pathophysiological mechanisms still remain unclear [33,34]. In terms of systemic vasodilatory effects occurring after anaesthetic agents and related accompanied mediators, studies have highlighted the role of NO, which plays an important role in vascular homeostasis [14,35]. However, the results of these studies are inconsistent. A study investigating the role of the endothelium in vasodilation induced by propofol and ST in isolated human mesenteric arteries has revealed that ST induces an NO-mediated endothelium-dependent relaxation response, whereas propofol induces an endothelium-independent relaxation response [10]. There are also in vitro studies in the literature suggesting that ST has no effect on vascular tone [25] and furthermore it decreases endothelium-dependent relaxation responses by inhibiting NOS [36,37]. However, propofol was reported to elicit an endothelium-derived hyperpolarised factor-mediated relaxation response in isolated human omental arteries and veins [11]. Experimental studies with in vitro and in vivo models have demonstrated that propofol activates NOS in leukocytes and endothelial cells and causes an increase in NO production, leading to a decrease in arterial pressure [26,38]. In fact, a recent study reported that the hypotensive effect of propofol may be more pronounced in eNOS polymorphisms due to the increased NO production [39]. In light of these studies, it can be concluded that NO is probably one of the mediators contributing to the hypotensive effects of both propofol and ST. The discrepancies observed in the outcomes of the studies may be attributed to the differences in methodology employed in the experimental procedures, the diversity of experimental animal models, and the variety of vascular beds utilised. It has been established since 2006 that human RBCs contain an active NOS enzyme and contribute significantly to homeostatic regulation by releasing NO into plasma, both in healthy and disease conditions [40]. Previous studies have shown that erythrocyte-derived NO plays a pivotal role in the regulation of blood pressure and blood flow [16,41,42]. Furthermore, a recent study reported that mice lacking the RBC-NOS enzyme exhibited a hypertensive response [43]. However, it remains unclear whether the stimulation of RBCs by anaesthetic agents such as ST and propofol increases NO production and if it has clinical significance in this regard. In this study, it was demonstrated that ST increased RBC-mediated NO production in a dose-dependent manner. Furthermore, the results of the experiments conducted in the presence of L-NAME demonstrated that NOS inhibition suppressed ST-mediated NO production in RBCs, thereby providing compelling evidence that RBC-NOS plays a pivotal role in NO-generating responses. It is well established that ST administration results in dose-dependent hypotension, which is attributed to peripheral vasodilation. The increase in NO production in RBCs at increasing ST doses, as observed in our study, suggests that RBC-derived NO may be responsible for the dose-dependent hypotensive effects of ST. Moreover, nitrite/nitrate concentrations in suspension medium increased after ST incubation, suggesting NO output from RBCs. In contrast, incubation of RBCs with propofol did not cause a significant difference in nitrite/nitrate levels measured in RBC suspensions. These results are consistent with intracellular NO concentration measurements obtained using flow cytometric method and confirm that ST, but not propofol, increased NO production in RBCs. These experiments were repeated with RBC suspensions containing both 25% and 40% haematocrit. Although our results showed that the nitrite/nitrate values measured in suspensions containing 40% haematocrit were higher than when it was 25%, there was no statistically significant difference between the two groups. NOS is constitutively expressed as a bi-domain enzyme comprised of N-terminal oxidase and reductase domains. Its activity is regulated by both domains via direct protein–protein interaction or phosphorylation [44]. Calcium-calmodulin binds to calmodulin binding sites on both domains, resulting in activation of the enzyme via displacement of auto-inhibitory loops. Thus, eNOS activity is proportional to the intracellular calcium level [44]. For this reason, we measured intracellular free Ca^+2^ level in RBCs in response to ST and propofol incubation. The results demonstrated an elevation in the intracellular free Ca^+2^ concentration of RBCs in response to ST exposure. In contrast, propofol did not alter the intracellular Ca^+2^ concentrations in RBCs. These results were consistent with the preceding findings of this study and indicate that an elevated intracellular Ca^+2^ level is a crucial factor in ST-induced activation of RBC-NOS. In contrast to the previous studies investigating leukocyte and endothelial cells, our results demonstrated that propofol did not contribute to NO production in RBCs [26,45]. The doses used in these studies were within the dose range we used in our study. The varying results may be attributed to the different activation mechanisms or cellular components of NOS enzymes across different cells. In light of the considerable body of evidence indicating that the vasodilatory effect of propofol is independent of NO, our findings lend support to the conclusion that NO is not a key player in the mechanism of action of propofol in RBCs. The results of this study clearly showed that ST increases NO production in RBCs in a dose-dependent manner and the produced NO is released into the suspension medium, providing the first data on the mechanism of action of ST in the literature. Nevertheless, further investigation is required to ascertain the clinical significance of this finding.

RBCs play an important role in regulating blood fluidity and viscosity with their unique mechanical behaviors of deformability and aggregation. In this study, the effects of ST and propofol on RBC mechanical behaviors were also investigated and it was found that neither of the molecules had any effect on deformability but they reduced the aggregation index. The ST effect on the aggregation index was more pronounced and caused a dose-dependent response. An in vitro study of the haemorheological effects of propofol demonstrated that there was no observable impact of propofol on RBC deformability and aggregation properties [46]. Nevertheless, no study evaluating the haemorrhagic effects of ST was identified in the literature. It has been demonstrated in previous studies that the introduction of a NO donor into the medium results in an improvement in the rheological properties of blood [47,48]. The dose-dependent decrease in RBC aggregation caused by ST is likely attributable to the fact that ST induces an increase in RBC-derived NO synthesis.

In recent years, the paradox of drug titration has come to the fore. The drug titration paradox occurs when higher drug concentrations are paradoxically associated with worse efficacy outcomes due to the titration of the individual’s drug dose to achieve the desired effect [17,18,19,20]. For instance, due to individual variability, some patients may require higher doses to achieve the desired effect, resulting in higher drug concentrations. However, this may lead to either the same or possibly worse clinical efficacy outcomes. Recent consensus has emerged that, at the population level, the observed correlation between drug concentration and outcome may not accurately represent the true relationship between drug concentration and its causal effect on outcome. Nevertheless, it must be acknowledged that such an approach may give rise to erroneous conclusions when evaluating the situation. It is imperative to recognise that correlation does not necessarily imply causality [49]. This underscores the critical importance of pharmacodynamic and pharmacokinetic studies in determining the optimal plasma concentration for achieving the desired pharmacological effects, while accounting for the intricacies of drug titration. In light of these considerations, a comprehensive dose range was investigated in the present study, considering the ongoing uncertainty surrounding the optimal plasma concentration for ST and propofol in clinical practice. In previous clinical studies, the 95% effective propofol concentration for loss of consciousness in patients was reported to be approximately 30 µM in the total fraction combining free and plasma-bound fractions [50,51,52]. However, in the majority of these studies, an opioid/sedative agent was administered in conjunction with the propofol to achieve this effect. In a study conducted by Kazama et al., the blood concentrations of propofol required to achieve a 50% reduction in responsiveness to stimulation for laryngoscopy, tracheal intubation with a laryngoscope, and tracheal intubation with a fibreoscope were reported to be 10.9, 19.6, and 19.9 µg/mL, respectively [24]. Additionally, it was observed that these concentrations were reduced by the administration of fentanyl. Accordingly, as propofol was employed as a sole agent in this investigation, the initial dose was established at 100 µM (17.8 µg/mL) and subsequent doses were examined. The administration of propofol did not result in any discernible impact on RBC-mediated NO production and release, irrespective of the dosage administered. Although ST is known to cause adverse effects such as haemodynamic dysfunction or immunosuppression at blood concentrations of 30–70 µg/mL, the relationship between serum concentrations and pharmacological efficacy has not been well characterised [22]. In the study of Becker et al. evaluating the therapeutic response based on ST plasma concentration in the induction of anaesthesia, it was reported that the total plasma level causing loss of corneal reflex and loss of trapezius muscle response was 39–42 µg/mL [53]. Hung et al. reported that ST plasma concentrations that prevented movement response with 50% probability were 15.6 ± 1 µg/mL for motor response to verbal command, 50.7 ± 2.9 µg/mL for laryngoscopy, and 78.8 ± 7.4 µg/mL for intubation [54]. A study investigating the pharmacokinetics of ST in patients with cerebral injury reported that a plasma ST concentration of 9–79 µg/mL resulted in deep sedation [55]. Prolonged infusion of ST shows non-linear kinetics, which may lead to increased plasma concentrations and prolonged apparent half-life. Due to Michaelis–Menten kinetics and hepatic enzyme autoinduction, intra- and inter-patient plasma concentrations and pharmacological effects may vary [21]. In a study by Stover et al., patients who had been receiving a long-term (mean 7 ± 3 days) ST infusion for increased intracranial pressure associated with traumatic brain injury were observed. It was found that the long-term infusion resulted in an increase in serum ST concentration reaching 130 µg/mL [23]. However, they also noted that serum ST levels are not a reliable indicator of pharmacological effects, such as the depth of anaesthesia. In light of the disparate doses and ST plasma concentrations documented in the extant literature, and the dearth of evidence assessing the pharmacological effects and drug toxicity, a broad range of doses was investigated, including 100 µM (26.4 µg/mL), 250 µM (66 µg/mL), 500 µM (132.2 µg/mL), and 1000 µM (264.3 µg/mL). It was shown that ST increased RBC-derived NO production at all doses and the EC50 was 415.8 ± 37.4 μM (109.9 ± 9.9 µg/mL). Despite the assertion that serum ST levels gauged in earlier investigations cannot be employed to forecast which patients will experience circulatory depression, ST may precipitate an augmentation in RBC-mediated NO production and release, thereby accounting for ST-induced dose-dependent hypotension in patients whose plasma serum levels attained the levels observed in our study. Nevertheless, in vivo studies are required to confirm this hypothesis.

Our study has limitations. Firstly, although the results of our study indicated that ST-mediated NO production and release in RBCs may contribute to hypotension, further investigation is required to confirm this hypothesis in vivo in diverse patient populations. Secondly, the impact of these agents on the vascular bed was not assessed in the present study. It would be beneficial to evaluate the contribution of RBC-derived NO to the vasodilatory effects of these anaesthetic agents using organ bath systems. Thirdly, it should be noted that the number of patients included in this study was relatively low. However, the sample size was sufficient to achieve adequate statistical power. In order to confirm the results of our study, it would be beneficial to conduct further research using an experimental model with ST and propofol on mice with and without RBC-NOS enzyme, given that our study was an in vitro study.

In conclusion, this study provides the first evidence of increased RBC-mediated NO production and release in response to ST administration. In contrast to the effects observed with ST, our results demonstrated that propofol had no impact on RBC-mediated NO production. Moreover, it was shown that both ST and propofol decreased the aggregation index and had no impact on RBC deformability.

## Figures and Tables

**Table 1:** Dosing with thiopental and propofol.

Doses	Sodium Thiopental Molecular Weight: 264.32 5 mg/50 µL (378 mM)	Propofol Molecular Weight: 178.271 10 mg/mL (56 mM)
1000 µM	264.3 µg/mL	178.3 µg/mL
500 µM	132.2 µg/mL	89.1 µg/mL
250 µM	66 µg/mL	44.6 µg/mL
100 µM	26.4 µg/mL	17.8 µg/mL

Description: doses under investigation in each sample.

**Table 2:** Patient characteristics. Data are presented as number (%) or median (IQR (min–max)). IQR, interquartile range.

Parameters	*n* = 12
Age, year	27.5 (25–30 (22–33))
Sex (male)	12 (100)
Height, cm	179 (177–181.5 (172–186))
Weight, kg	80 (79–84.5 (77–90))
Body mass index, kg/m^2^	25.2 (24.5–26.5 (24.3–27.4))

## Data Availability

All data generated or analysed during this study are included in this article. Further inquiries can be directed to the corresponding author.

## List of Abbreviations

RBC	Erythrocyte
NO	Nitric oxide
ASA	American Society of Anesthesiologists
Ca^+2^	Calcium
ST	Sodium thiopental
NOS	Nitric oxide synthase
DAF-FM-DA	4-amino-5-methylamino-20,70-difluorofluorescein diacetate
EI	Elongation index
SS1/2	Half-maximal erythrocyte deformation
AI	Aggregation index
L-NAME	L-N-acetyl-methyl-arginine
SEM	Standard error of the mean

## References

[1] Skibiski J., Patel P., Abdijadid S. (2025). Barbiturates.

[2] Khan K.S., Hayes I., Buggy D.J. (2014). Pharmacology of anaesthetic agents I: Intravenous anaesthetic agents. Contin. Educ. Anaesth. Crit. Care Pain.

[3] Holtkamp M. (2018). Pharmacotherapy for Refractory and Super-Refractory Status Epilepticus in Adults. Drugs.

[4] Ryu J.A., Jung W., Jung Y.J., Kwon D.Y., Kang K., Choi H., Kong D.S., Seol H.J., Lee J.I. (2019). Early prediction of neurological outcome after barbiturate coma therapy in patients undergoing brain tumor surgery. PLoS ONE.

[5] Green D.W. (2015). Cardiac output decrease and propofol: What is the mechanism?. Br. J. Anaesth..

[6] Goodchild C.S., Serrao J.M. (2015). Propofol-induced cardiovascular depression: Science and art. Br. J. Anaesth..

[7] Peck T., Harris B. (2021). Pharmacology for Anaesthesia and Intensive Care.

[8] Wang L., Wu B., Sun Y., Xu T., Zhang X., Zhou M., Jiang W. (2010). Translocation of protein kinase C isoforms is involved in propofol-induced endothelial nitric oxide synthase activation. Br. J. Anaesth..

[9] Wang Y., Zhou H., Wu B., Zhou Q., Cui D., Wang L. (2015). Protein Kinase C Isoforms Distinctly Regulate Propofol-induced Endothelium-dependent and Endothelium-independent Vasodilation. J. Cardiovasc. Pharmacol..

[10] Moreno L., Martinez-Cuesta M.A., Muedra V., Beltran B., Esplugues J. (1997). Role of the endothelium in the relaxation induced by propofol and thiopental in isolated arteries from man. J. Pharm. Pharmacol..

[11] Bodelsson G., Sandstrom K., Wallerstedt S.M., Hidestal J., Tornebrandt K., Bodelsson M. (2000). Effects of propofol on substance P-induced relaxation in isolated human omental arteries and veins. Eur. J. Anaesthesiol..

[12] Forstermann U., Sessa W.C. (2012). Nitric oxide synthases: Regulation and function. Eur. Heart J..

[13] Oliveira-Paula G.H., Lacchini R., Tanus-Santos J.E. (2017). Clinical and pharmacogenetic impact of endothelial nitric oxide synthase polymorphisms on cardiovascular diseases. Nitric Oxide.

[14] Toda N., Toda H., Hatano Y. (2007). Nitric oxide: Involvement in the effects of anesthetic agents. Anesthesiology.

[15] Oliveira-Paula G.H., Pinheiro L.C., Ferreira G.C., Garcia W.N.P., Lacchini R., Garcia L.V., Tanus-Santos J.E. (2018). Angiotensin converting enzyme inhibitors enhance the hypotensive effects of propofol by increasing nitric oxide production. Free Radic. Biol. Med..

[16] Gajecki D., Gawrys J., Szahidewicz-Krupska E., Doroszko A. (2022). Role of Erythrocytes in Nitric Oxide Metabolism and Paracrine Regulation of Endothelial Function. Antioxidants.

[17] Schnider T.W., Minto C.F., Filipovic M. (2021). The Drug Titration Paradox: Correlation of More Drug with Less Effect in Clinical Data. Clin. Pharmacol. Ther..

[18] Goulooze S.C., Krekels E.H.J., Knibbe C.A.J., van Noort M. (2025). The Drug Titration Paradox in the Presence of Intra-Individual Variation: Can we Estimate the True Concentration-Effect Relationship?. AAPS J..

[19] Schnider T.W., Minto C.F., Luginbuhl M., Egan T.D. (2022). The drug titration paradox: More drug does not correlate with more effect in individual clinical data. Br. J. Anaesth..

[20] Egan T.D. (2022). The drug titration paradox: Something obvious finally understood. Br. J. Anaesth..

[21] Huynh F., Mabasa V.H., Ensom M.H. (2009). A critical review: Does thiopental continuous infusion warrant therapeutic drug monitoring in the critical care population?. Ther. Drug Monit..

[22] Dabricot E., Seqat I., Dailler F., Rheims S., Boulogne S., Balanca B. (2021). How to monitor thiopental administration in the intensive care unit for refectory status epilepticus or intracranial hypertension?. Crit. Care.

[23] Stover J.F., Lenzlinger P.M., Stocker R., Morganti-Kossmann M.C., Imhof H.G., Trentz O., Kossmann T. (1998). Thiopental in CSF and serum correlates with prolonged loss of cortical activity. Eur. Neurol..

[24] Kazama T., Ikeda K., Morita K., Katoh T., Kikura M. (1998). Propofol concentration required for endotracheal intubation with a laryngoscope or fiberscope and its interaction with fentanyl. Anesth. Analg..

[25] Park W.K., Lynch C., Johns R.A. (1992). Effects of propofol and thiopental in isolated rat aorta and pulmonary artery. Anesthesiology.

[26] Wang L., Jiang W. (2010). Propofol induces endothelial nitric oxide synthase phosphorylation and activation in human umbilical vein endothelial cells by inhibiting protein kinase C delta expression. Eur. J. Anaesthesiol..

[27] Hardeman M.R., Dobbe J.G., Ince C. (2001). The Laser-assisted Optical Rotational Cell Analyzer (LORCA) as red blood cell aggregometer. Clin. Hemorheol. Microcirc..

[28] Baskurt O.K., Meiselman H.J. (2004). Analyzing shear stress-elongation index curves: Comparison of two approaches to simplify data presentation. Clin. Hemorheol. Microcirc..

[29] Pace N.L., Stylianou M.P. (2007). Advances in and limitations of up-and-down methodology: A precis of clinical use, study design, and dose estimation in anesthesia research. Anesthesiology.

[30] Walsh M., Devereaux P.J., Garg A.X., Kurz A., Turan A., Rodseth R.N., Cywinski J., Thabane L., Sessler D.I. (2013). Relationship between intraoperative mean arterial pressure and clinical outcomes after noncardiac surgery: Toward an empirical definition of hypotension. Anesthesiology.

[31] Saugel B., Sessler D.I. (2021). Perioperative Blood Pressure Management. Anesthesiology.

[32] Jor O., Maca J., Koutna J., Gemrotova M., Vymazal T., Litschmannova M., Sevcik P., Reimer P., Mikulova V., Trlicova M. (2018). Hypotension after induction of general anesthesia: Occurrence, risk factors, and therapy. A prospective multicentre observational study. J. Anesth..

[33] Hino H., Matsuura T., Kihara Y., Tsujikawa S., Mori T., Nishikawa K. (2019). Comparison between hemodynamic effects of propofol and thiopental during general anesthesia induction with remifentanil infusion: A double-blind, age-stratified, randomized study. J. Anesth..

[34] Kakazu C.Z., Lippmann M. (2015). Playing with fire: Debate about propofol-induced hypotension. Br. J. Anaesth..

[35] Gragasin F.S., Davidge S.T. (2009). The effects of propofol on vascular function in mesenteric arteries of the aging rat. Am. J. Physiol. Heart Circ. Physiol..

[36] Roh W.S., Ding X., Murray P.A. (2006). Propofol and thiopental attenuate adenosine triphosphate-sensitive potassium channel relaxation in pulmonary veins. Am. J. Physiol. Lung Cell Mol. Physiol..

[37] Castillo C., Escalante B., Terron J.A., Valencia I., Castillo E.F. (1997). Effects of thiopental on endothelium-dependent responses in rat aorta. Arch. Med. Res..

[38] De La Cruz J.P., Paez M.V., Carmona J.A., De La Cuesta F.S. (1999). Antiplatelet effect of the anaesthetic drug propofol: Influence of red blood cells and leucocytes. Br. J. Pharmacol..

[39] Oliveira-Paula G.H., Lacchini R., Pinheiro L.C., Ferreira G.C., Luizon M.R., Garcia W.N.P., Garcia L.V., Tanus-Santos J.E. (2018). Endothelial nitric oxide synthase polymorphisms affect the changes in blood pressure and nitric oxide bioavailability induced by propofol. Nitric Oxide.

[40] Kleinbongard P., Schulz R., Rassaf T., Lauer T., Dejam A., Jax T., Kumara I., Gharini P., Kabanova S., Ozuyaman B. (2006). Red blood cells express a functional endothelial nitric oxide synthase. Blood.

[41] Basarici I., Ozen N., Kilavuz E., Kisak F., Basrali F., Yaras N., Koksoy S., Celik M.L., Ulker P. (2020). Concealed role of red blood cells in pathogenesis of pulmonary arterial hypertension: Decreased red blood cell nitric oxide generation and effect of Rho-Kinase inhibitor fasudil. Clin. Hemorheol. Microcirc..

[42] Helms C.C., Gladwin M.T., Kim-Shapiro D.B. (2018). Erythrocytes and Vascular Function: Oxygen and Nitric Oxide. Front. Physiol..

[43] Leo F., Suvorava T., Heuser S.K., Li J., LoBue A., Barbarino F., Piragine E., Schneckmann R., Hutzler B., Good M.E. (2021). Red Blood Cell and Endothelial eNOS Independently Regulate Circulating Nitric Oxide Metabolites and Blood Pressure. Circulation.

[44] Fleming I. (2010). Molecular mechanisms underlying the activation of eNOS. Pflug. Arch..

[45] Gonzalez-Correa J.A., Cruz-Andreotti E., Arrebola M.M., Lopez-Villodres J.A., Jodar M., De La Cruz J.P. (2008). Effects of propofol on the leukocyte nitric oxide pathway: In vitro and ex vivo studies in surgical patients. Naunyn Schmiedeberg Arch. Pharmacol..

[46] Kim Y.H., Chung H.G., Myung S.A., Rha J.H., Yang S., Nam M.H., Shin S.H., Lim C.H. (2012). In vitro effect of clinical propofol concentrations on red blood cell aggregation and deformability. Clin. Hemorheol. Microcirc..

[47] Bor-Kucukatay M., Yalcin O., Gokalp O., Kipmen-Korgun D., Yesilkaya A., Baykal A., Ispir M., Senturk U.K., Kaputlu I., Baskurt O.K. (2000). Red blood cell rheological alterations in hypertension induced by chronic inhibition of nitric oxide synthesis in rats. Clin. Hemorheol. Microcirc..

[48] Alexy T., Detterich J., Connes P., Toth K., Nader E., Kenyeres P., Arriola-Montenegro J., Ulker P., Simmonds M.J. (2022). Physical Properties of Blood and their Relationship to Clinical Conditions. Front. Physiol..

[49] Minto C.F., Egan T.D., Schnider T.W. (2024). Drug Titration Paradox: An Emerging Concept in Clinical Pharmacology. Anesthesiology.

[50] Smith C., McEwan A.I., Jhaveri R., Wilkinson M., Goodman D., Smith L.R., Canada A.T., Glass P.S. (1994). The interaction of fentanyl on the Cp50 of propofol for loss of consciousness and skin incision. Anesthesiology.

[51] Li S., Yu F., Zhu H., Yang Y., Yang L., Lian J. (2016). The median effective concentration (EC50) of propofol with different doses of fentanyl during colonoscopy in elderly patients. BMC Anesth..

[52] Dawidowicz A.L., Kalitynski R., Kobielski M., Pieniadz J. (2006). Influence of propofol concentration in human plasma on free fraction of the drug. Chem. Biol. Interact..

[53] Becker K.E. (1978). Plasma levels of thiopental necessary for anesthesia. Anesthesiology.

[54] Hung O.R., Varvel J.R., Shafer S.L., Stanski D.R. (1992). Thiopental pharmacodynamics. II. Quantitation of clinical and electroencephalographic depth of anesthesia. Anesthesiology.

[55] Russo H., Simon N., Duboin M.P., Urien S. (1997). Population pharmacokinetics of high-dose thiopental in patients with cerebral injuries. Clin. Pharmacol. Ther..

